# One-year post-acute COVID-19 syndrome and mortality in South Korea: a nationwide matched cohort study using claims data

**DOI:** 10.3389/fpubh.2024.1403153

**Published:** 2024-07-10

**Authors:** Jung-Hyun Won, Yesol Hong, Siun Kim, Howard Lee

**Affiliations:** ^1^Department of Molecular Medicine and Biopharmaceutical Sciences, Graduate School of Convergence Science and Technology, Seoul National University, Seoul, Republic of Korea; ^2^Center for Convergence Approaches in Drug Development, Graduate School of Convergence Science and Technology, Seoul National University, Seoul, Republic of Korea; ^3^Biomedical Research Institute, Seoul National University Hospital, Seoul, Republic of Korea; ^4^Department of Clinical Pharmacology and Therapeutics, Seoul National University Hospital, Seoul, Republic of Korea; ^5^Advanced Institute of Convergence Technology, Suwon, Republic of Korea

**Keywords:** COVID-19, post-acute sequelae of COVID-19, mortality, South Korea, cohort studies

## Abstract

**Background:**

Current understanding of post-COVID-19 syndrome in South Korea is primarily based on survey studies or research targeting specific patient groups, such as those hospitalized. Moreover, the majority of relevant studies have been conducted in European and North American populations, which may limit their applicability to the South Korean context. To address this gap, our study explores the one-year outcomes of COVID-19, focusing on the potential post-acute syndrome and all-cause mortality in South Korea.

**Methods:**

This retrospective cohort study used nationwide claims data in South Korea, including adults aged >18 with records between January 20, 2020, and February 25, 2021. Patients were classified into COVID-19 and non-COVID-19 groups and matched 1:1 based on propensity scores. Primary outcomes were 12-month post-acute COVID-19 syndrome and all-cause mortality.

**Results:**

The study involved 34,802 matched patients. The COVID-19 group had significantly elevated risks of coagulopathies (OR = 2.70 [2.24, 3.28]; *p* < 0.001), chronic lower respiratory diseases (OR = 1.96 [1.80, 2.14]; *p* < 0.001), symptoms of the circulatory and respiratory systems (OR = 1.91 [1.80, 2.04]; *p* < 0.001), mood disorders (OR = 1.67 [1.51, 1.86]; *p* < 0.001), cardiac diseases (OR = 1.39 [1.21, 1.59]; *p* < 0.001), and symptoms of cognition, perception, emotional state, and behavior (OR = 1.15 [1.04, 1.27]; *p* = 0.005). All-cause mortality was higher in the COVID-19 group during the 6 months (OR = 1.34 [1.06, 1.69]; *p* = 0.015), but gradually decreased, reaching an OR of 0.996 ([0.83, 1.19]; *p* = 0.964) at 1 year.

**Conclusion:**

In South Korea, the 12-month post-acute COVID-19 syndrome includes coagulopathies, respiratory issues, mood disorders, and cardiac diseases. The risk of all-cause mortality post-COVID-19 is heightened for up to 6 months, then significantly decreases and resolves within a year.

## 1 Introduction

Post-acute COVID-19 Syndrome is characterized by a range of new, recurring, or persistent symptoms or conditions that COVID-19 survivors experience beyond the acute phase (1). The prevalence of post-acute COVID-19 syndrome ranges from 5 to 50%, depending on factors such as the definition used, the population studied, and the time period observed (2). Health complications associated with post-acute COVID-19 Syndrome include, but are not limited to, thromboembolic disorders, neuropsychiatric issues, and chronic fatigue syndrome (3).

Although progress has been made in understanding the long-term effects of COVID-19 up to one-year post-infection, knowledge gaps still remain. For example, previous investigations have been studied in specific patient groups such as hospitalized, or were conducted predominantly in European and American countries, with relatively few studies focusing on Asian populations, especially in South Korea (4–7). Furthermore, much of the existing research in Asia focused only on subjective findings based on survey or narrowly targeted specific medical conditions (8–12).

Motivated by the knowledge gap, we investigated the one-year consequences of COVID-19, focusing on the potential post-acute COVID-19 syndrome and all-cause mortality in South Korea. Leveraging the wide coverage of a nationwide population-based claims database in South Korea (13–15), we aim to understand how COVID-19 affects the general population across all demographics and varying COVID-19 severities. To this end, we identified diseases, symptoms and all-cause mortality experienced by individuals who contracted COVID-19 *prior to* the initiation of the COVID-19 vaccination in South Korea. We then evaluated whether the diseases, symptoms and all-cause mortality happened more frequently or less in individuals who were infected with COVID-19 than in those not in the same period.

## 2 Materials and methods

### 2.1 Data source

We used a nationwide claims database named the HIRA Covid-19 OMOP database, provided by the Health Insurance Review & Assessment Service (HIRA) in South Korea, standardized according to the Observational Medical Outcomes Partnership Common Data Model (OMOP CDM, version 5.3) (13). Maintained by the governmental institute in South Korea, the HIRA Covid-19 OMOP database contains information about COVID-19 diagnosis, managed by the Korea Disease Control and Prevention Agency, and all-cause mortality data, linked with the national death registry of Statistics Korea (13). All COVID-19 diagnoses during our study period were confirmed only by reverse transcription polymerase chain reaction (RT-PCR) testing (13).

This study was approved by the Institutional Review Board (IRB) of Seoul National University Hospital (SNUH), Seoul, South Korea. Due to the retrospective and de-identified nature of this study, the SNUH IRB waived the requirement for obtaining informed consent from study participants (IRB No: E-2207-022-1337).

### 2.2 Study population

Eligible patients were adults aged >18 years old and had at least one visit record in the HIRA CDM database between January 20, 2020 and February 25, 2021. These two dates were the day when the first case of COVID-19 was confirmed in South Korea and the day when the COVID-19 vaccination program started in South Korea, respectively. Eligible patients were divided into two groups based on the presence or absence of a COVID-19 diagnosis record during the study period: *COVID-19* and *non-COVID-19 groups*, respectively.

The index date was the date of the first recorded COVID-19 diagnosis or the initial visit date within the study period for the COVID-19 and the non-COVID-19 groups, respectively. To ensure covariate balance, eligible patients were matched 1:1 based on propensity scores derived from a logistic regression model incorporating age, sex, Charlson Comorbidity Index (CCI), and index month/year. Baseline covariates were matched using data from 1 year prior to the index date to account for pre-existing conditions. We utilized the OHDSI adaptation of the CCI, which employs SNOMED CT coding and has been validated across major studies for its comparable performance to the Quan adaptation. Propensity score matching was performed using the open-source OHDSI Cohort Method packages in R (16).

### 2.3 Outcomes

The primary outcomes of interest were the potential 12-month post-acute COVID-19 syndrome, defined as the occurrence of pre-specified diseases and symptoms (Supplementary Table S1) observed between one-month and a year after the index date, and all-cause mortality within a year after the index date. Pre-specified diseases and symptoms were chosen based on their possible association with post-acute COVID-19 syndrome (3). We categorized potential 12-month post-acute COVID-19 syndrome according to the Korean Standard Classification of Diseases and Causes of Death, 8th edition (KCD-8). Outcomes related to external causes (e.g., injury, poisoning) or congenital anomalies were excluded from the pre-specified diseases and symptoms. The temporal trends in the primary outcomes were also assessed over a year divided into three periods: the acute phase (between the index date and 1 month after an index date), the 6-month post-acute phase (between 1 and 6 months after an index date), and the 12-month post-acute phase (between 1 and 12 months after an index date).

### 2.4 Statistical analyses

We estimated an odds ratio (OR) of the primary outcomes between the COVID-19 and non-COVID-19 groups using a multiple logistic regression, which incorporated age at the index date, sex, CCI, and index month/year as covariates. Kaplan–Meier Survival curves were used to visually compare the differences in survival probability between the two groups. Furthermore, we analyzed temporal trends in ORs for the primary outcomes using linear regression, where OR and numerically encoded time periods were the dependent and independent variables, respectively. All statistical analyses were performed using R (version 3.5.1; R Foundation, Vienna, Austria).

## 3 Results

### 3.1 Study population

A total of 18,278 and 5,501,604 patients initially met the eligibility criteria for the COVID-19 and non-COVID-19 groups, respectively. After 1:1 propensity score matching, the study population consisted of 34,802 patients with both the COVID-19 and non-COVID-19 groups accounting for half of the total patients (i.e., *n* = 17,401) (Table 1). Baseline characteristics were adequately balanced, with the average age of the study population at 49 years, and females constituting 48%. The average Charlson Comorbidity Index (CCI) score in the COVID-19 group was 1.65, slightly lower than that in the non-COVID-19 group (1.768).

**Table 1:** Baseline characteristics of the COVID-19 and non-COVID-19 groups before and after propensity score matching.

	Before PS matching (overall group)			After PS matching^*^		
	COVID-19 (*N* = 18,278)	Non-COVID-19 (*N* = 5,501,604)	aSD	COVID-19 (*N* = 17,401)	Non-COVID-19 (*N* = 17,401)	aSD
Age, years	50.0	51.2	0.068	48.8	48.6	0.016
Charlson Comorbidity Index	1.923	1.523	0.194	1.650	1.768	0.055
**Sex (%)**						
Male	47.4%	49.5%	0.043	47.6%	47.5%	0.003
Female	52.6%	50.5%	0.043	52.4%	52.5%	0.003
**Index date (%)**						
February 2020–May 2020	18.0%	47.8%	0.298	18.9%	20.1%	0.012
June 2020–September 2020	17.3%	49.6%	0.323	18.0%	17.8%	0.020
October 2020–February 2021	66.8%	5.3%	0.615	65.3%	64.3%	0.010

Values are numbers (percentages) unless stated otherwise. PS, propensity score; aSD, absolute standardized difference.

*Balance in covariate distribution between two groups was assessed using the absolute standardized mean difference.

### 3.2 12-month post-acute COVID-19 syndrome

**Figure 1:**
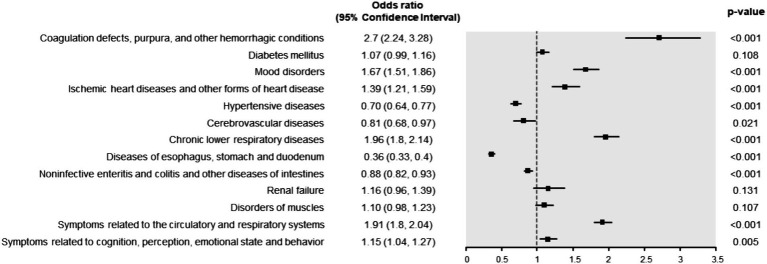
Odds ratio (OR) of 12-month post-acute COVID-19 syndrome. An OR greater than one indicates a higher risk of post-acute COVID-19 syndrome in the COVID-19 group than in the non-COVID-19 group.

The COVID-19 group had significantly higher risks of coagulation defects, purpura, and other hemorrhagic conditions (OR = 2.70 [2.24, 3.28]; *p* < 0.001), chronic lower respiratory diseases (OR = 1.96 [1.80, 2.14]; *p* < 0.001), symptoms related to the circulatory and respiratory systems (OR = 1.91 [1.80, 2.04], *p* < 0.001), mood disorders (OR = 1.67 [1.51, 1.86]; *p* < 0.001), ischemic heart diseases and other forms of heart disease (OR = 1.39 [1.21, 1.59]; *p* < 0.001), and symptoms related to cognition, perception, emotional state and behavior (OR = 1.15 [1.04, 1.27]; *p* = 0.005) (Figure 1). Conversely, the risks of noninfective enteritis and colitis (OR = 0.88 [0.82, 0.93]; *p* < 0.001), cerebrovascular diseases (OR = 0.81 [0.68, 0.97]; *p* < 0.001), hypertensive disorders (OR = 0.70 [0.64, 0.77]; *p* < 0.001), and diseases of esophagus, stomach, and duodenum (OR = 0.36 [0.33, 0.40]; *p* < 0.001) were significantly lower in the COVID-19 group than in the non-COVID-19 group. On the other hand, the risks of diabetes mellitus (OR = 1.07 [0.99, 1.16]; *p* = 0.108), muscular disorders (OR = 1.10 [0.98, 1.23]; *p* = 0.107), and renal failure (OR = 1.16 [0.96, 1.39]; *p* = 0.131) did not differ significantly between the two groups.

### 3.3 All-cause mortality

**Figure 2:**
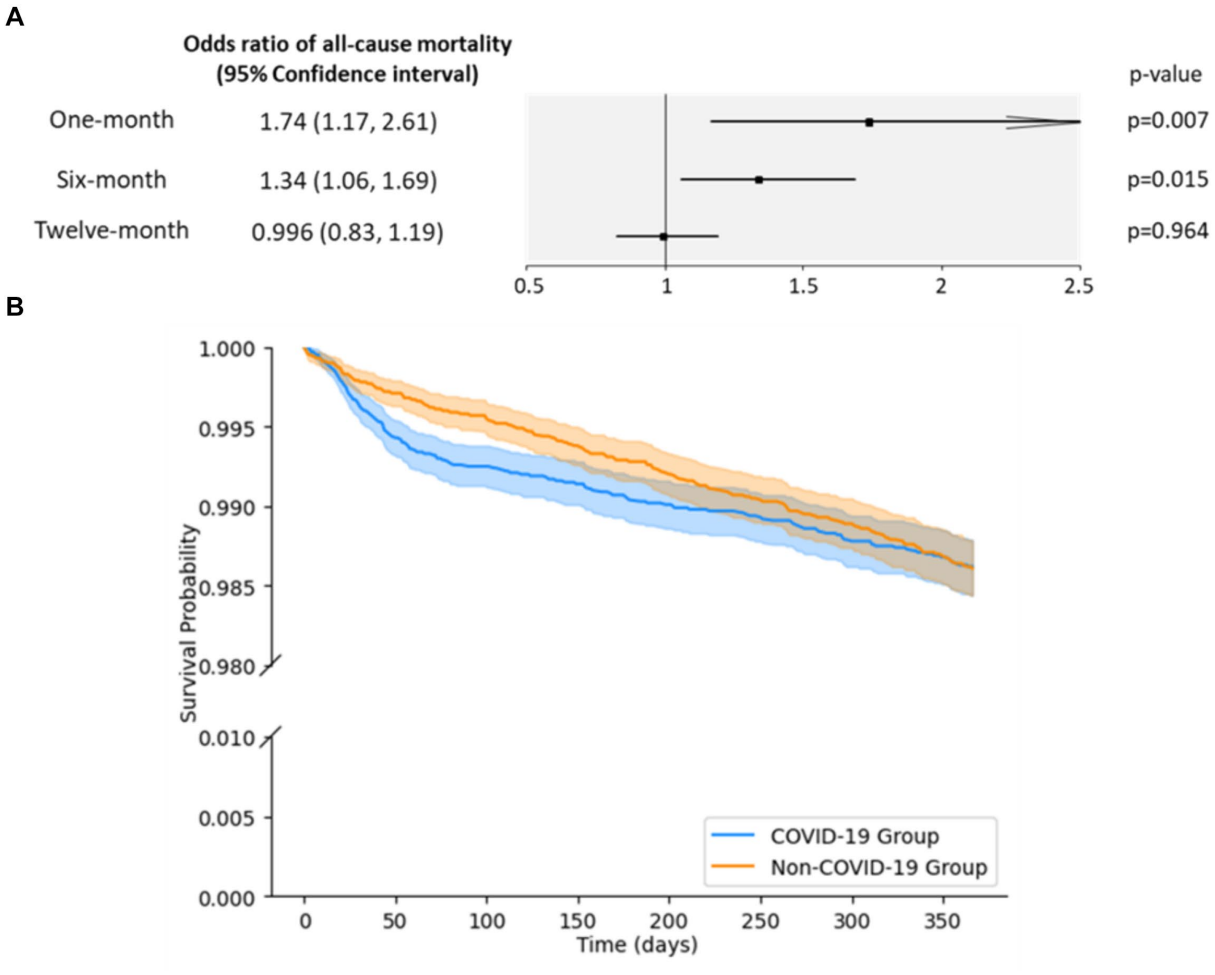
Odds ratio (OR) and survival probabilities for all-cause mortality. **(A)** ORs of all-cause mortality by period. An OR greater than one indicates a higher risk of all-cause mortality in the COVID-19 group than in the non-COVID-19 group. **(B)** Kaplan–Meier survival curves for all-cause mortality. The curves represent the survival probabilities in days for the COVID-19 group (blue) and the non-COVID-19 group (orange). The shaded regions correspond to the lower and upper 95% confidence bounds.

In the acute phase, the COVID-19 group had significantly higher odds of all-cause mortality than the non-COVID-19 group (OR = 1.74 [1.17, 2.61]; *p* = 0.007, Figure 2A). However, this increased risk disappeared by a year after the index date (OR = 0.996 [0.83, 1.19]; *p* = 0.964), with a statistically significant 0.6-fold decrease in the ORs over a year (Supplementary Table S3). While the COVID-19 group had a slightly lower survival probability than the non-COVID-19 group (Figure 2B), this difference was not statistically significant and the largest observed difference in survival probability between the two groups was 0.33 percentage points at day 82.

### 3.4 Temporal changes in post COVID-19 syndrome

**Figure 3:**
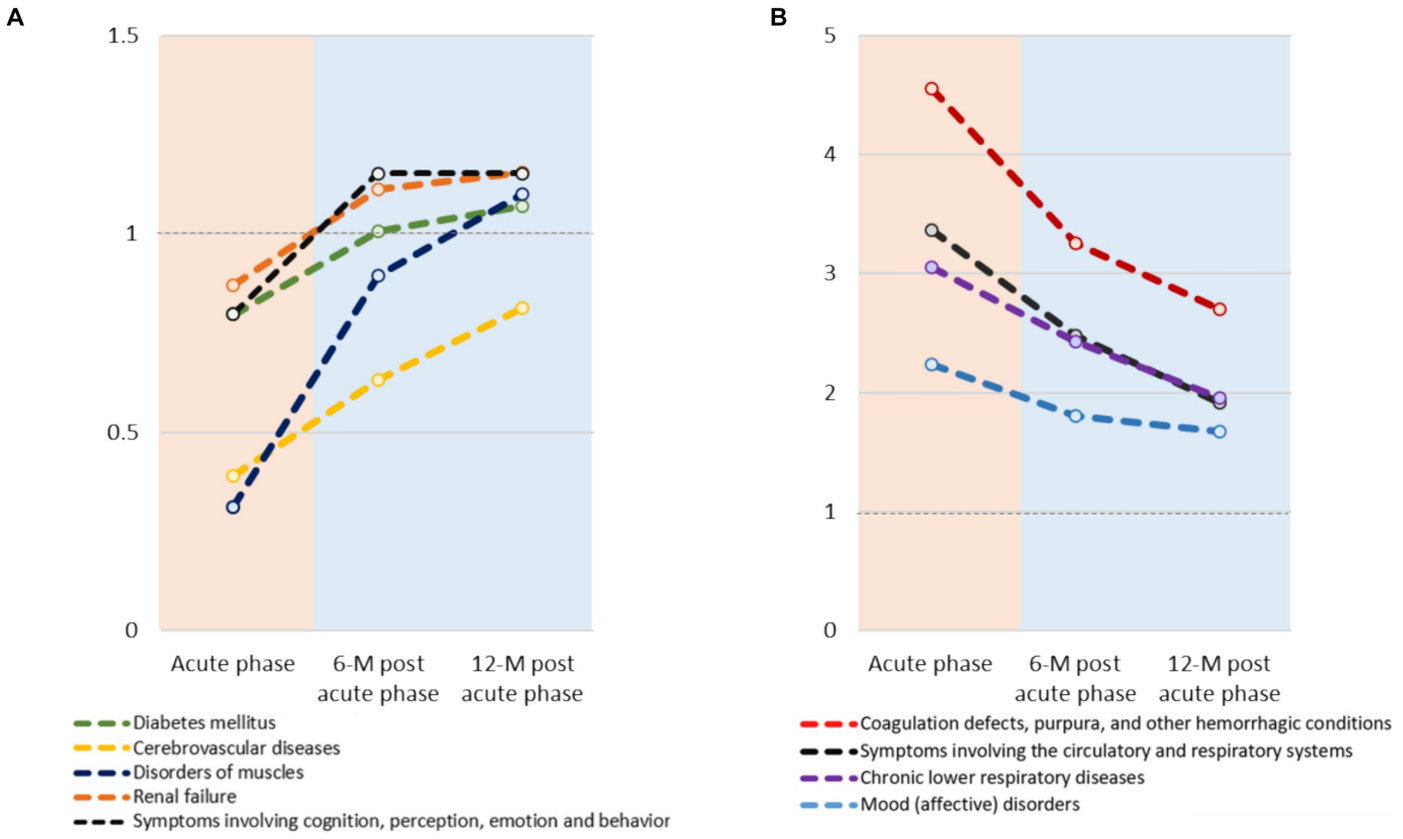
Temporal trends in odds ratios (OR) of post COVID-19 syndrome over a year between COVID-19 and non-COVID-19 groups: **(A)** increasing trend and **(B)** decreasing trend. Only those with a difference of OR ≥ 0.2 between the acute and 12-month post-acute phases, and a fold change in OR of ≥1.2 or ≤0.7 between these phases are shown.

Although statistically not significant, consistent increases in the ORs were noted for disorders of muscles (3.5-fold increase), cerebrovascular diseases (2.1-fold increase), symptoms related to cognition, perception, emotional state and behavior (1.4-fold increase), diabetes mellitus (1.3-fold increase) and renal failure (1.3-fold increase) (Figure 3; Supplementary Table S3). Conversely, a significant decrease in OR was observed for chronic lower respiratory diseases (0.6-fold decrease). Furthermore, consistent but statistically insignificant decreases in the ORs for mood disorders (0.7-fold decrease), coagulation defects, purpura, and other hemorrhagic conditions (0.6-fold decrease).

## 4 Discussion

Using a nationwide claims database in South Korea, we identified several diseases that were more frequently experienced by patients contracted with COVID-19 than those not, which could be collectively referred to as post-acute COVID-19 syndrome. Notably, the COVID-19 group had a significantly higher risk of developing coagulation defects, purpura, and other hemorrhagic conditions for a year post-infection (OR = 2.70 [2.24, 3.28], *p* < 0.001). A probable mechanism for the heightened risk of hemorrhagic conditions is the cytokine storm triggered by the SARS-CoV-2 virus (17–21). Furthermore, the interaction of SARS-CoV-2 with endothelial cells in the lung, particularly those with overexpressed angiotensin-converting enzyme 2 (ACE2), could exacerbate the risk of coagulopathies by inducing a pro-coagulative and inflammatory state (17–21). Moreover, we noted a significantly higher risk of ischemic heart diseases and other forms of heart diseases in the COVID-19 group (OR = 1.39 [1.21, 1.59], *p* < 0.001). Our findings suggest that the impact of SARS-CoV-2 on hematological and inflammatory functions extends beyond the acute phase, with potential long-term implications, including the development of cardiovascular diseases such as ischemic heart diseases (22–24).

A significantly elevated risk of chronic lower respiratory diseases (OR = 1.96 [1.80, 2.14], all *p* < 0.001) was observed in COVID-19 patients, potentially exacerbated by the virus-induced inflammatory environment, which may accelerate the progression and worsen symptoms of pre-existing conditions such as chronic obstructive pulmonary disease (COPD) (25–28). These findings underscore the long-term respiratory implications of COVID-19, contributing to the emergence of new chronic respiratory diseases and aggravating existing conditions. Likewise, there was a sustained increase in the risk of mood disorders among COVID-19 patients for up to a year (OR = 1.67 [1.51, 1.86], *p* < 0.001). Factors such as pandemic-induced stress, uncertainty, and strict quarantine measures, including social distancing and isolation, highlight the necessity for comprehensive, long-term mental health care strategies for COVID-19 survivors (29–31).

Moreover, COVID-19 patients had a higher risk of all-cause mortality during both the acute phase (OR = 1.74 [1.17, 2.61], *p* = 0.007) and at 6 months (OR = 1.34 [1.06, 1.69], *p* = 0.015), which diminished by 1 year (OR = 0.996 [0.83, 1.19], *p* = 0.964). Supporting these observations, another study demonstrated that the COVID-19 pandemic did not significantly affect the overall national mortality rate in South Korea (32). Furthermore, South Korea has maintained a low COVID-19 death rate at 0.7 (per 100,000) since the start of the pandemic (0.2 since May 2020; 0.2 since June 2020), which was much lower than that in other countries such as the United States, i.e., 60.3 since the start of the pandemic; 36.9 since May 2020; 27.2 since June 2020 (33). Several factors contribute to this low mortality rate, including South Korea’s robust public health preparedness, effective management protocols, and the demographic characteristics of our study population.

South Korea was ranked as the fifth best country globally for disaster preparedness and management protocols aimed to reduce COVID-19 mortality (34). In addition, the government provided COVID-19 diagnoses and treatments free of charge to all COVID-19 patients supporting patient recovery (29, 35). These comprehensive healthcare and public health strategies in South Korea may have mitigated the long-term mortality risks associated with COVID-19. Moreover, the demographic characteristics of the study population, with an average age of 48.8 years and a moderate comorbidity burden (CCI score of 1.65), may have influenced the observed all-cause mortality. While these factors collectively contribute to our findings, the precise reasons for the low mortality in COVID-19 patients in South Korea remain unclear.

However, the risk for diseases of the digestive system remained significantly lower in the COVID-19 group over a year than in the non-COVID-19 group (noninfective enteritis and colitis, OR = 0.88 [0.82, 0.93]); diseases of esophagus, stomach, and duodenum (OR = 0.36 [0.33, 0.40]; all *p* < 0.001) in the COVID-19 group than in the non-COVID-19 group. This observation may be attributed to the paradoxical dual role of the ACE2 receptor in the digestive system, where its overexpression increases susceptibility to SARS-CoV-2 but its anti-inflammatory effects could potentially protect against severe digestive complications (36, 37). Additionally, many physicians in South Korea often prescribe gastrointestinal medications such as rebamapide and famotidine along with other drugs, particularly in patients with common respiratory illness (38–40). This prescription practice, which became more widespread after the South Korean government began covering the full cost of medications for COVID-19 patients (40), could have contributed to the lower risks for diseases of the digestive system in the COVID-19 group in our findings. On the other hand, several studies have proposed potential therapeutic advantages of gastrointestinal medications as COVID-19 treatments (41–43). However, the direct impact of these medications on digestive diseases in COVID-19 patients remains unclear.

This study had two major limitations. First, potential confounders such as socioeconomic status and vaccination status that could have affected health outcomes were not fully adjusted (35). However, we adjusted for various factors to minimize the impact of potential confounders. Additionally, by using nationwide data, our study ensured a broad and representative sample. Furthermore, we included only those study participants whose index dates were before the start of the COVID-19 vaccination program in South Korea (35). In addition, it is unlikely, if not impossible, that our study population included patients vaccinated during the follow-up period. Although the COVID-19 vaccination program in South Korea began on February 26, 2021, its roll-out has been seriously hampered by the failure in securing a sufficient number of vaccine doses to cover the population until the end of 2021. Therefore, the early vaccination program in South Korea was strictly prioritized to people over 70 years old. Additionally, those confirmed with COVID-19 (all testing results were PCR-based) were excluded from the vaccination program at least until mid-2022. Given the average age of our study population was 50 and the one-year follow-up period ends February 2022, it is unlikely, if not impossible, that our study population included patients vaccinated during the follow-up period.

Secondly, the utilization of claims data may have introduced bias in disease reporting and diagnosis. The claims database in South Korea includes primary and additional diagnoses that are recorded as the main reasons for treatment or prescription. However, claims data often prioritize specific diagnoses for billing purposes, potentially overlooking other health issues. This issue was particularly pronounced during the COVID-19 pandemic, when there was heightened attention on reporting severe respiratory illnesses and commonly prioritized illnesses related to COVID-19. Consequently, this may have led to an underreporting of less severe or secondary conditions not closely associated with COVID-19, skewing our understanding of the prevalence and diversity of health conditions (44–46).

We observed a slightly lower risk of hypertensive disorders (OR = 0.70 [0.64, 0.77], *p* < 0.001) and cerebrovascular diseases (OR = 0.81 [0.68, 0.97], *p* = 0.021) in the COVID-19 group compared to the non-COVID-19 group. This finding differs from other studies that reported insignificant associations between COVID-19 and these conditions (47–49). This discrepancy may have been caused by the underreporting or deprioritization of those conditions by physicians in South Korea, particularly during the peak of the COVID-19 pandemic.

Additionally, changes in public behavior during the COVID-19 pandemic may explain the lower odds ratios for certain diseases. For example, healthcare utilization for hypertension increased among the general population in South Korea during the pandemic (50, 51). This suggests that while COVID-19 patients might have experienced delays in care for certain conditions, non-COVID-19 patients continued to seek and receive care, possibly to address health concerns proactively before any healthcare service disruptions could occur. Therefore, the lower risk of certain conditions in the COVID-19 patients observed in this study should not be misconstrued as a protective effect of COVID-19. Instead, it is more likely to reflect changes in healthcare utilization and physician reporting patterns during the pandemic, which differently impacted access to healthcare and the management of COVID-19 and non-COVID-19 patients. Conversely, diseases that showed an increased risk in the COVID-19 group may have actually had a lower risk, influenced by similar biases.

While we used comprehensive list of predefined diseases and symptoms covered a wide range of health conditions to reduce the risk of missing less common health issues, further studies using electronic medical records (EMR), which provide a more accurate and details of patient health status and clinical outcomes with physician-confirmed diagnoses and laboratory details, may further validate our findings. Such studies could also address potential biases introduced by the prioritization of specific diagnoses during the pandemic.

In conclusion, post-acute COVID-19 syndrome in South Korea comprises coagulopathies, lower respiratory diseases, mood disorders, and ischemic heart diseases. All-cause mortality is also increased after infection with COVID-19 for up to 6 months, after which the risk significantly decreases and eventually resolves within a year. However, these results should be interpreted with caution, considering the changes in healthcare delivery and reporting biases specific to the Korean healthcare system during the pandemic.

## Data availability statement

This study was conducted using anonymized data in a retrospective analysis, adhering to the guidelines set by the Institutional Review Board (IRB). In compliance with IRB regulations, the anonymized data used in this study cannot be shared. This restriction is in place to uphold the privacy and confidentiality standards required by the IRB.

## Ethics statement

The studies involving humans were approved by the Institutional Review Board (IRB) of Seoul National University Hospital (SNUH), Seoul, South Korea (IRB No: E-2207-022-1337). The studies were conducted in accordance with local legislation and institutional requirements. Written informed consent for participation was not required from the participants or their legal guardians/next of kin because the database was fully anonymized.

## Author contributions

J-HW: Conceptualization, Data curation, Formal analysis, Funding acquisition, Investigation, Methodology, Project administration, Visualization, Writing – original draft. YH: Conceptualization, Data curation, Formal analysis, Investigation, Methodology, Project administration, Writing – review & editing, Funding acquisition. SK: Conceptualization, Investigation, Methodology, Validation, Writing – review & editing. HL: Conceptualization, Funding acquisition, Investigation, Methodology, Supervision, Writing – review & editing.
