# Supplementary material for: One-year post-acute COVID-19 syndrome and mortality in South Korea: a nationwide matched cohort study using claims data
Source: Front Public Health. 2024 Jul 10;12:1403153. doi: 10.3389/fpubh.2024.1403153 (PMC11266063; doi:10.3389/fpubh.2024.1403153)
Supplement: Supplementary file 1 [file Table_1.DOCX]

**SUPPLEMENTARY**

**Supplementary Table 1.** Pre-specified diseases and symptoms categorized according to the Korean Standard Classification of Diseases and Causes of Death, 8th edition.

| **Diseases and Symptoms** | **ICD-10 Codes^*^** | **OMOP Common Data Model Concept ID** |
| --- | --- | --- |
| Coagulation defects, purpura, and other hemorrhagic conditions | D65–D69 | 432585, 432863, 432869, 432870, 436093, 437241, 441259, 441264, 4179872, 40321716 |
| Diabetes mellitus | E10–E14 | 192279, 200687, 201254, 201820, 201826, 318712, 321822, 376065, 377821, 435216, 439770, 442793, 443412, 443727, 443729, 443730, 443731, 443732, 443733, 443734, 443735, 443767, 4008576, 4096041, 4096042, 4096670, 4096671, 4099652, 4193704, 4221933, 4224419, 4224879, 4327944, 40480000, 42538169 |
| Mood disorders | F30–F39 | 433440, 433991, 434911, 435220, 435226, 436665, 439254, 439256, 440078, 440383, 440696, 444100, 4077577, 4098302, 4150985, 4228802, 4250023, 4282316, 4307111, 4310821, 4327337, 4333677, 4333678, 4336957, 35610112, 35622934 |
| Ischemic heart diseases and other forms of heart disease | I20–I25; I30–I52 | 258449, 312327, 312653, 313791, 313792, 314054, 314059, 314370, 314379, 314383, 314658, 314666, 315286, 315293, 315296, 315564, 316135, 316139, 316428, 316998, 316999, 317576, 318448, 318772, 318773, 319038, 319835, 319844, 319845, 320116, 320127, 320425, 320744, 320746, 321042, 321315, 321318, 321319, 321320, 321588, 432937, 434376, 434663, 438168, 438170, 438171, 438172, 439279, 439846, 440736, 441589, 443962, 4029831, 4057008, 4068155, 4088351, 4089462, 4090739, 4091901, 4103295, 4108215, 4108217, 4108218, 4108219, 4108220, 4108677, 4108678, 4108679, 4108680, 4108805, 4108814, 4108817, 4108819, 4108950, 4110937, 4110961, 4111099, 4111412, 4111413, 4111414, 4111552, 4114159, 4115173, 4119460, 4119953, 4120088, 4124683, 4124693, 4124694, 4127089, 4134889, 4138837, 4141360, 4143969, 4154290, 4158911, 4163710, 4166844, 4176969, 4181182, 4185572, 4189343, 4190773, 4203625, 4209011, 4217075, 4231274, 4232337, 4232697, 4237062, 4237202, 4250169, 4252872, 4261842, 4268046, 4270024, 4275423, 4295336, 4317150, 4331309, 36712986, 36714994, 43020480, 44784217, 45769461 |
| Hypertensive diseases | I10–I15 | 195556, 201313, 314378, 317895, 319034, 319826, 320128, 439694, 439695, 439696, 442604, 443771, 443919, 4110948, 4118993, 4289933 |
| Cerebrovascular diseases | I60–I69 | 312938, 316437, 376713, 380747, 381316, 381591, 432923, 434056, 436430, 443454, 4027461, 4043731, 4045749, 4049659, 4108356, 4108952, 4110185, 4110186, 4110189, 4110190, 4110192, 4111708, 4111714, 4111715, 4111720, 4111721, 4112026, 4148906, 4159164, 4179912, 4353709, 37016924, 42535424, 42535425, 42535426, 42538062, 42539269, 43530623, 43530674, 43530727, 45766199 |
| Chronic lower respiratory diseases | J40–J47 | 255573, 255841, 256449, 256451, 257004, 257905, 261325, 261889, 313236, 317009, 4110051, 4110056, 4112826, 4142738, 4143828, 4145356, 4145497, 4163244, 4177944, 4191479, 4193588, 4196712, 4209097, 4212099, 4286497, 37116845, 46269770, 46269776, 46269784 |
| Diseases of esophagus, stomach and duodenum | K20–K31 | 22820, 22955, 25844, 26441, 30437, 30753, 31610, 31884, 134681, 192667, 193249, 194071, 194987, 195300, 195301, 195306, 199866, 201340, 201619, 316457, 318186, 318800, 433515, 433516, 434085, 443344, 4000609, 4006994, 4027663, 4027729, 4046500, 4056933, 4057513, 4057953, 4059178, 4066036, 4101104, 4101870, 4138962, 4144111, 4144112, 4146517, 4147683, 4150681, 4163865, 4164920, 4169592, 4173408, 4174044, 4177387, 4194543, 4195231, 4198381, 4204555, 4209746, 4211001, 4217947, 4222896, 4224926, 4225273, 4231580, 4232181, 4247008, 4248429, 4265479, 4265600, 4274491, 4280942, 4289526, 4289830, 4291028, 4291649, 4294973, 4296611, 4303233, 4336230, 4344497, |
| Noninfective enteritis and colitis and other diseases of intestines | K50–K52; K55–K64 | 75576, 75580, 75860, 76725, 77317, 78799, 79061, 80141, 81064, 81336, 81893, 134404, 192357, 192674, 192953, 193239, 193242, 193252, 193518, 193807, 194684, 195002, 195585, 196151, 196160, 197024, , 197328, 197593, 197603, 197925, 198465, 198475, 199064, 200773, 201606, 201618, 201894, 201900, 201905, 442190, 443327, 4006305, 4009163, 4026004, 4043371, 4055116, 4055201, 4055202, 4057381, 4057822, 4057826, 4057835, 4092161, 4105587, 4134562, 4134603, 4145825, 4152372, 4164898, 4197094, 4213865, 4216644, 4228686, 4234788, 4235753, , 4261072, 4262720, 4266809, 4272162, 4285898, 4287929, 4326601, 4340811, 4341633, 4341635, 4342660, 36715881, 36715898, 36715916, 36715917, 36715918, 36716700, 36717183, 37016128, 37116441, 37119299, 40482241, 40482865, 40483171, 40488439, 42536652, 45763562, 45772085, 46269901, 46269907, 46270529, 46272242, 46273183 |
| Disorders of muscles | M60–M63 | 72413, 73001, 73854, 75048, 76508, 77076, 77653, 78236, 137275, 4001124, 4001125, 4001626, 4001627, 4001654, 4002808, 4002809, 4002813, 4080368, 4109087, 4112302, 4147680, 4168796, 4196401, 4208264, 4345577, 4347291, 37117206, 37396333, 40490403, 46270387, 46270388, 46270389, 46270390, 46270415, 46273515, 46273643 |
| Renal failure | N17–N19 | 192359, 193782, 197320, 197329, 198185, 432961, 443597, 443601, 443611, 443612, 443614, 45757466, 46271022 |
| Symptoms and signs involving the circulatory and respiratory systems | R00–R09 | 314754, 261687, 194475, 4169095, 439928, 254761, 78786, 259153, 315531, 312437, 316822, 444070, 4262562, 317109, 4328356, 433596, 4180628, 4114164, 4305080, 4042140, 253321, 4298207, 314171, 4208719, 315078, 4168213, 316814, 134159, 4132926, 4305577, 4096682, 4317284, 4145848, 77670, 141693 |
| Symptoms and signs involving cognition, perception, emotional state, and behavior | R40–R46 | 4195585, 4168683, 4229448, 436817, 433031, 4085332, 43530714, 380834, 4268911, 4185711, 4229897, 4269314, 4164151, 4025215, 4251913, 4024707, 4168212, 46270715, 4152296, 4216219, 376961, 4168684, 439147, 4168682, 4164648, 4168681, 4152347, 4169106, 4171906, 433316, 4293175, 4275889, 436235, 4171905, 4198081 |

^*^Outcomes related to external causes (e.g., injury, poisoning) or congenital anomalies were excluded from the pre-specified diseases and symptoms.

**Supplementary Table 2.** Odds ratio (OR) of post COVID-19 syndrome. An OR above 1 indicates a higher risk in the COVID-19 group.

|  | **OR** | **CI95lb** | **CI95ub** | **p-value** |
| --- | --- | --- | --- | --- |
| **Coagulation defects, purpura, and other hemorrhagic conditions** |  |  |  |  |
| Acute phase | 4.556 | 3.333 | 6.366 | <0.001 |
| 6-month post-acute phase | 3.250 | 2.558 | 4.174 | <0.001 |
| 12-month post-acute phase | 2.699 | 2.235 | 3.280 | <0.001 |
| **Diabetes mellitus** |  |  |  |  |
| Acute phase | 0.796 | 0.703 | 0.901 | <0.001 |
| 6-month post-acute phase | 1.006 | 0.912 | 1.110 | 0.900 |
| 12-month post-acute phase | 1.071 | 0.985 | 1.163 | 0.108 |
| **Mood disorders** |  |  |  |  |
| Acute phase | 2.234 | 1.869 | 2.681 | <0.001 |
| 6-month post-acute phase | 1.803 | 1.590 | 2.047 | <0.001 |
| 12-month post-acute phase | 1.674 | 1.508 | 1.860 | <0.001 |
| **Ischemic heart diseases and other forms of heart disease** |  |  |  |  |
| Acute phase | 1.204 | 0.965 | 1.506 | 0.102 |
| 6-month post-acute phase | 1.238 | 1.046 | 1.466 | 0.013 |
| 12-month post-acute phase | 1.385 | 1.208 | 1.589 | <0.001 |
| **Hypertensive diseases** |  |  |  |  |
| Acute phase | 0.650 | 0.569 | 0.742 | <0.001 |
| 6-month post-acute phase | 0.701 | 0.630 | 0.779 | <0.001 |
| 12-month post-acute phase | 0.699 | 0.639 | 0.765 | <0.001 |
| **Cerebrovascular diseases** |  |  |  |  |
| Acute phase | 0.390 | 0.287 | 0.522 | <0.001 |
| 6-month post-acute phase | 0.633 | 0.509 | 0.783 | <0.001 |
| 12-month post-acute phase | 0.812 | 0.681 | 0.968 | 0.021 |
| **Chronic lower respiratory diseases** |  |  |  |  |
| Acute phase | 3.051 | 2.678 | 3.486 | <0.001 |
| 6-month post-acute phase | 2.433 | 2.194 | 2.700 | <0.001 |
| 12-month post-acute phase | 1.960 | 1.797 | 2.138 | <0.001 |
| **Diseases of esophagus, stomach and duodenum** |  |  |  |  |
| Acute phase | 0.229 | 0.196 | 0.265 | <0.001 |
| 6-month post-acute phase | 0.322 | 0.287 | 0.361 | <0.001 |
| 12-month post-acute phase | 0.361 | 0.328 | 0.398 | <0.001 |
| **Noninfective enteritis and colitis and other diseases of intestines** |  |  |  |  |
| Acute phase | 0.820 | 0.743 | 0.905 | <0.001 |
| 6-month post-acute phase | 0.877 | 0.814 | 0.945 | <0.001 |
| 12-month post-acute phase | 0.875 | 0.823 | 0.931 | <0.001 |
| **Disorders of muscles** |  |  |  |  |
| Acute phase | 0.311 | 0.226 | 0.422 | <0.001 |
| 6-month post-acute phase | 0.896 | 0.769 | 1.044 | 0.161 |
| 12-month post-acute phase | 1.099 | 0.980 | 1.234 | 0.107 |
| **Renal failure** |  |  |  |  |
| Acute phase | 0.872 | 0.661 | 1.147 | 0.328 |
| 6-month post-acute phase | 1.113 | 0.893 | 1.389 | 0.340 |
| 12-month post-acute phase | 1.155 | 0.959 | 1.392 | 0.131 |
| **Symptoms and signs involving the circulatory and respiratory systems** |  |  |  |  |
| Acute phase | 3.364 | 3.053 | 3.714 | <0.001 |
| 6-month post-acute phase | 2.477 | 2.296 | 2.674 | <0.001 |
| 12-month post-acute phase | 1.911 | 1.795 | 2.035 | <0.001 |
| **Symptoms and signs involving cognition, perception, emotional state and behavior** |  |  |  |  |
| Acute phase | 0.799 | 0.653 | 0.976 | 0.029 |
| 6-month post-acute phase | 1.153 | 1.016 | 1.310 | 0.028 |
| 12-month post-acute phase | 1.152 | 1.044 | 1.273 | 0.005 |

OR, odds ratio; CI95lb, 95% Confidence Interval Lower Bound; CI95ub, 95% Confidence Interval Upper Bound.

**Supplementary Table 3.** Results of linear regression models to investigate the temporal Trends in odds ratios (ORs) for post COVID-19 syndrome. The slope indicates the trend direction and magnitude over a year (positive slope for increasing trends, negative slope for decreasing trends). The p-value indicates the statistical significance of each trend, with values in bold and underlined to highlight statistically significant trends (p < 0.05). The 'R-squared' value indicates the proportion of variance in ORs explained by the model.

|  | **Slope** | **p-value** | **R-squared** |
| --- | --- | --- | --- |
| Coagulation defects, purpura, and other hemorrhagic conditions | −0.929 | 0.147 | 0.948 |
| Diabetes mellitus | 0.137 | 0.188 | 0.915 |
| Mood disorders | −0.280 | 0.192 | 0.912 |
| Ischemic heart diseases and other forms of heart disease | 0.090 | 0.220 | 0.885 |
| Hypertensive diseases | 0.024 | 0.355 | 0.719 |
| Cerebrovascular diseases | 0.211 | 0.056 | 0.992 |
| Chronic lower respiratory diseases | −0.546 | **0.049** | 0.994 |
| Diseases of esophagus, stomach and duodenum | 0.066 | 0.148 | 0.947 |
| Noninfective enteritis and colitis and other diseases of intestines | 0.027 | 0.353 | 0.723 |
| Disorders of muscles | 0.394 | 0.174 | 0.927 |
| Renal failure | 0.141 | 0.246 | 0.859 |
| Symptoms and signs involving the circulatory and respiratory systems | −0.727 | 0.081 | 0.984 |
| Symptoms and signs involving cognition, perception, emotional state, and behavior | 0.176 | 0.335 | 0.748 |
| All-cause mortality | −0.370 | **0.030** | 0.998 |
